# Three-Dimensional Imaging and Histopathological Features of Third Metacarpal/Tarsal Parasagittal Groove and Proximal Phalanx Sagittal Groove Fissures in Thoroughbred Horses

**DOI:** 10.3390/ani13182912

**Published:** 2023-09-14

**Authors:** Szu-Ting Lin, Alastair K. Foote, Nicholas M. Bolas, Vanessa G. Peter, Rachel Pokora, Hayley Patrick, David R. Sargan, Rachel C. Murray

**Affiliations:** 1 Department of Veterinary Medicine, University of Cambridge, Madingley Rd., Cambridge CB3 0ES, UK; drs20@cam.ac.uk; 2 Rossdales Veterinary Surgeons, Cotton End Rd., Exning, Newmarket CB8 7NN, UK; alastair.foote@rossdales.com (A.K.F.); vanessa.peter@rossdales.com (V.G.P.); rachel.pokora@rossdales.com (R.P.); rachel.murray@rossdales.com (R.C.M.); 3 Hallmarq Veterinary Imaging, Unit 5 Bridge Park, Merrow Lane, Guildford GU4 7BF, UK; nick.bolas@hallmarq.net; 4 Swayne and Partners Veterinary Surgeons, Western Way, Bury St Edmunds IP33 3SP, UK; patrick.hayley@sky.com

**Keywords:** fissure, computed tomography, cone beam, fan beam, magnetic resonance imaging, fatigue injury, stress fracture

## Abstract

**Simple Summary:**

Fractures of the third metacarpal/tarsal parasagittal groove and proximal phalanx sagittal groove are common in racehorses. It is important to detect precursor pathologies including fissures to prevent the propagation to fracture. This study aims to identify the imaging features and compare the diagnosis of fissures on cone-beam (CB) computed tomography (CT), fan-beam (FB) CT, and low-field magnetic resonance imaging (MRI) to histopathology associated with fissures. Fissures were characterised on CBCT and FBCT as hypoattenuating linear defects, striated hypoattenuating lines, or subchondral irregularity. Fissures were characterised on MRI as subchondral hypo-/hyperintense defects. The diagnostic sensitivity was highest in CBCT, followed by FBCT and MRI, while specificity was highest in MRI, followed by FBCT and CBCT. Fissures identified on CT were associated with histopathology of subchondral bone sclerosis, microcracks, and collapse. In conclusion, all modalities were able to identify fissures with sensitivity higher in CT and specificity higher in MRI. CT-identified fissures were associated with histopathological indications of fatigue bone injuries. Imaging features and histopathological features of fissures characterised in this study may help clinical identification and image interpretation of fissures in horses.

**Abstract:**

Fissure in the third metacarpal/tarsal parasagittal groove and proximal phalanx sagittal groove is a potential prodromal pathology of fracture; therefore, early identification and characterisation of fissures using non-invasive imaging could be of clinical value. Thirty-three equine cadaver limbs underwent standing cone-beam (CB) computed tomography (CT), fan-beam (FB) CT, low-field magnetic resonance imaging (MRI), and macro/histo-pathological examination. Imaging diagnoses of fissures were compared to microscopic examination. Imaging features of fissures were described. Histopathological findings were scored and compared between locations with and without fissures on CT. Microscopic examination identified 114/291 locations with fissures. The diagnostic sensitivity and specificity were 88.5% and 61.3% for CBCT, 84.1% and 72.3% for FBCT, and 43.6% and 85.2% for MRI. Four types of imaging features of fissures were characterised on CT: (1) CBCT/FBCT hypoattenuating linear defects, (2) CBCT/FBCT striated hypoattenuated lines, (3) CBCT/FBCT subchondral irregularity, and (4) CBCT striated hypoattenuating lines and FBCT subchondral irregularity. Fissures on MRI appeared as subchondral bone hypo-/hyperintense defects. Microscopic scores of subchondral bone sclerosis, microcracks, and collapse were significantly higher in locations with CT-identified fissures. All imaging modalities were able to identify fissures. Fissures identified on CT were associated with histopathology of fatigue injuries.

## 1. Introduction

Parasagittal condylar fracture of the third metacarpal/metatarsal bone and sagittal fracture of the proximal phalanx are the most common fractures in Thoroughbred racehorses [1,2]. These fractures have been considered as stress/fatigue fractures resulting from repetitive microdamage and remodelling of the subchondral bone, accumulation of microcracks in the calcified cartilage, and propagation of microcracks into fissures in the subchondral bone [3,4,5]. Fissures are defined here as osteochondral interface defects located in the third metacarpal/tarsal parasagittal groove and proximal phalanx sagittal groove. Previous pathology studies found linear defects in the articular cartilage, calcified cartilage, and subchondral bone at locations commonly affected by stress fractures, which had histological features of fatigue injuries including microcracks in the calcified cartilage and subchondral bone plate, and subchondral bone sclerosis and microdamage [3,4,5,6]. It is therefore considered important to identify prodromal features of stress fractures to potentially prevent catastrophic injuries [7,8,9,10,11,12,13].

Imaging investigation of fractures has shown a superiority of three-dimensional imaging techniques such as computed tomography (CT) and magnetic resonance imaging (MRI) in identifying fissures and the associated osseous pathology compared to radiography [8,10,11,14,15,16,17]. Radiography is known to have limited sensitivity for identifying fissures, which appeared as subtle radiolucent lines in the subchondral bone due to structural superimposition on images [3,8,9,11,14,15,18]. On CT images, third metacarpal/metatarsal parasagittal groove fissures and proximal phalanx sagittal groove incomplete fractures have been characterised as hypoattenuating, linear defects in the subchondral bone with surrounding sclerosis [3,8,14,16,19,20]. CT technology is reported to have advantages in identifying fissures and assessing details of structural changes but lacks some information about the pathological status of fissures [14,15]. On MRI, features of fissures reported include linear hyperintensity in the third metacarpal/tarsal parasagittal groove and the proximal phalanx sagittal groove with surrounding bone hypointensity. Signal intensity patterns reflecting fluid on fluid-sensitive sequences in the subchondral/trabecular bone may be considered a marker of increased risk for potential fracture [10,11,18,19,21].

As standing CT and MR imaging become more available [22,23,24,25], the assessment of these advanced imaging modalities in the detection of fissures and a better understanding of the associated pathology are essential for clinical management. The aims of this study were to (1) describe the distribution and imaging features of third metacarpal/tarsal parasagittal groove and proximal phalanx sagittal groove fissures on images acquired using a standing CBCT, a 64-slice FBCT, and a standing low-field MRI system; (2) compare the identification of fissures between each modality by using microscopic findings of microcracks in the calcified cartilage and subchondral bone plate as a gold standard [6,26,27]; and (3) assess whether histopathological features of fatigue injuries, including subchondral bone sclerosis, microcracks, remodelling, and collapse, were associated with fissures identified on images [6,28]. It was hypothesised that (1) both CT systems are superior to the MRI in identifying fissures, with the FBCT superior to the CBCT; (2) MRI is able to identify pathological features in the surrounding bone relevant to the status of fissures; and (3) there are histopathological features of fatigue injuries associated with detected fissures.

## 2. Materials and Methods

### 2.1. Materials

The study was approved by the University of Cambridge Ethical Review committee (project number: CR558).

In total, 33 cadaver limbs (17 forelimbs and 16 hindlimbs) from 10 Thoroughbred horses aged 1–11 years were used in this study. Nine of the horses had been raced or were in race-training. All horses were euthanised for reasons other than the study: severe proximal interphalangeal joints osteoarthritis (1), proximal phalanx incomplete fracture (1), proximal phalanx complete fracture (1), condylar complete fracture (1 lateral condyle and 1 medial condyle), head injury (1), pelvic injury (1), severe osteochondral lesion (1), proximal sesamoid bone fracture (1), and pneumonia (1). Cadaveric limbs were collected at the carpus or tarsus level within 8 h of euthanasia. Cadaver limbs were assigned an individual code, but the identity of the case was not revealed to the image interpreter until all of the image analyses had been completed.

A standing low field MRI system (Hallmarq sMRI, Hallmarq Veterinary Imaging Ltd., Surrey, United Kingdom) ^a^, a standing CBCT (Hallmarq slCT, Hallmarq Veterinary Imaging Ltd., Guildford, United Kingdom) ^b^, and a 64-slice FBCT (Aquilion 64, Canon Medical Systems, Tochigi, Japan) ^c^ system were used to acquire CT and MR images of the metacarpo/metatarsophalangeal region.

### 2.2. Methods

#### 2.2.1. Handling of Cadaver Limbs for Image Acquisition

Images were acquired with either MRI or CT acquisition first, followed by the other imaging modality:

(1) MR images were acquired using a low-field (0.27 T) open magnet MRI system within 8 h of cadaver limb collection. After MR imaging, the limb was frozen at −20 °C for 8 h in a frame to standardise the imaging position between MRI and CT [29]. CBCT and FBCT images were then acquired from the frozen limb.
(2) The cadaver limb was frozen at −20 °C for at least 8 h in the frame immediately after collection to standardise the imaging position between MRI and CT. CBCT and FBCT images were acquired from the frozen limb. After CT imaging, the frozen limb was then defrosted in the frame for at least 8 h for MR imaging.

#### 2.2.2. Standing Low-Field MRI

The cadaver limb was held in the frame for MR imaging to simulate a standing position. MR images were acquired using 2 sets of MRI sequences [30]:

(1) MRI-1: Sequences selected for optimal image quality but longer acquisition time: T1-weighted (W) 3D high resolution (HR), T2*W 3D, T2W fast spin echo (FSE), short tau inversion recovery (STIR) FSE, proton density (SE) spin echo (SE), and T1W SE sequences;
(2) MRI-2: Sequences selected for a shorter acquisition time: T1W gradient echo (GRE) FAST, T2*W GRE FAST, T2W fast spin echo (FSE) FAST, and STIR FSE FAST sequences, to simulate a combination more often used clinically.

Sagittal, dorsal, and transverse MR images of all sequences were obtained in the following planes:

(1) Sagittal: aligned parallel to the longitudinal axis of the third metacarpus/tarsus and the proximal phalanx and perpendicular to the articular surface;
(2) Dorsal: aligned parallel to the dorsal aspects of the third metacarpus/tarsus and the proximal phalanx;
(3) Transverse: aligned perpendicular, 30° clockwise, and 30° anti-clockwise to the longitudinal axis of the third metacarpus/tarsus and proximal phalanx.

#### 2.2.3. Standing Cone-Beam CT (CBCT)

The frozen limb was taped on the CBCT platform in a standing position with the metacarpo/metatarsophalangeal region placed at the centre of the field of view (20 cm × 20 cm). Two fiducial markers were taped proximal and distal to the metacarpo/metatarsophalangeal joint on the lateral side to reflect their use for motion correction in a clinical scan. During image acquisition, the X-ray generator and detector rotated 360° synchronously around the metacarpo/metatarsophalangeal joint. Acquisition variables were 75 kV and 0.7 mA, 60 s acquisition time, and 900 projections. Data were reconstructed into a 800 × 800 × 800 voxel matrix with 0.25 mm thickness.

#### 2.2.4. Fan-Beam CT (FBCT)

The frozen limb was placed parallel to the FBCT platform for image acquisition. Acquisition parameters were 120 kV and 350 mA, 6–10 s acquisition time. Data were reconstructed into a 512 × 512 pixel matrix with 0.5 mm thickness.

#### 2.2.5. Image Analysis

The CBCT, FBCT, MRI-1, and MRI-2 images were all assessed in a different order by quasi-randomisation of the identity codes. The images were assessed twice by a trained interpreter (PhD student) and overseen by recognised imaging specialists (R.C.M. and V.G.P.). The first and second interpretation were at least 4 months apart.

**Figure 1:**
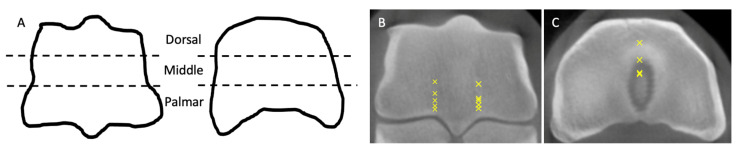
Locations for image analysis and locations observed with fissures on image analysis. The third metacarpal/tarsal parasagittal groove and the proximal phalanx sagittal groove were divided into dorsal, middle, and palmar/plantar locations by the dorsal and palmar/plantar borders of the collateral fossae ((**A**); dotted lines). Locations with fissures observed on CT and MR images were tracked for the collection of bone specimens ((**B**,**C**); marks)).

On CBCT, FBCT, and MR images, the third metacarpal/tarsal parasagittal groove and the proximal phalanx sagittal groove were divided into dorsal, middle, and palmar/plantar locations, separated by the dorsal and palmar/plantar borders of the collateral fossae (Figure 1).

All locations on CT and MR images were assessed in sagittal, dorsal, and transverse planes for (1) the presence of fissures at the dorsal, middle, and palmar/plantar locations of the third metacarpal/tarsal parasagittal groove and proximal phalanx sagittal groove; (2) the features of fissures on CBCT, FBCT, and MR images, including the previously reported hypoattenuating linear defects on CT images and hyperintense linear defects on MR images, and additional observed features; and (3) adjacent osseous abnormality including STIR hyperintensity on MR images.

Longitudinal length of fissures was measured on CBCT and FBCT images reconstructed in a dorsal plane aligned parallel to the dorsal aspect of the third metacarpus/tarsus.

The locations where fissures were identified were noted for later collection of bone specimens for histological examination. To determine the locations, two transverse images, at the level of the collateral fossa of the third metacarpus/tarsus and at the level of the palmar eminence of the proximal phalanx, were recorded for each cadaver limb, and locations with fissures identified were marked on the transverse images (Figure 1).

#### 2.2.6. Macroscopic Examination

Digital photographs were taken of the articular surfaces of the third metacarpal/metatarsal bone and the proximal phalanx. The third metacarpal/tarsal parasagittal groove and proximal phalanx sagittal groove were examined for previously reported features of fissures: linear erosions, cracks, and defects of articular cartilage and subchondral bone and subchondral bone discolouration [3,4,7,8,14].

#### 2.2.7. Histological Preparation

Dorsal sections of bone specimens were collected at the dorsal, middle, and palmar/plantar locations of the third metacarpal/tarsal parasagittal groove and proximal phalanx sagittal groove in each cadaver limb. Locations with fissures observed on CT and MR images were also collected based on the transverse image record (Figure 1).

Bone specimens at each location were collected with a band saw, fixed in 10% neutral buffered formalin, decalcified, and paraffin-embedded. Sections (4–5 μm thickness) were stained with Haematoxylin and Eosin (H&E) and Toluidine blue.

#### 2.2.8. Histological Examination

The histological slides were examined under a light microscope and a polarised light microscope by a trained interpreter (PhD student) and overseen by recognised specialists (A.K.F. (MA, VetMB, PhD, FRCPath, MRCVS) and R.C.M. (MA, VetMB, MS, PhD, Dip ACVS, Assoc ECVDI, MRCVS)). Microscopic findings of microcracks in the calcified cartilage and subchondral bone plate, characterised by a single to multiple oblique, linear or coalescing defects extending from the calcified cartilage into subchondral bone plate, were considered as fissures. These histological findings were considered the gold standard for verifying CT and MR imaging identification of fissures [6,26,27].

Histopathological changes associated with imaging-identified fissures were assessed in three regions: (1) hyaline cartilage, (2) calcified cartilage, and (3) subchondral/trabecular bone regions based on a previously reported scoring system [28]. The scoring of histopathological changes ranged from 0 (no abnormality) to 3 (severe histopathology), and the scores were compared between CT-identified fissures and non-fissures. In the scoring system, subchondral bone sclerosis, microcracks, and collapse were considered as the histopathological features of fatigue injuries [6,28].

To verify MRI findings of increased fluid signal in bone, additional observation of proteinaceous exudate and haemorrhage in the subchondral/trabecular region was recorded (absence/presence).

### 2.3. Data Analysis

Statistical analyses used IBM SPSS Statistics 28.0.0 software. The sensitivity and specificity with 95% Confidence intervals (CI95) of CBCT, FBCT, and MRI systems in identifying fissures were analysed using the second imaging reading data by cross tabulation of imaging diagnoses and microscopic findings of microcracks in the calcified cartilage and subchondral bone plate as the gold standard. The agreement between imaging modalities and the gold standard, CBCT and FBCT agreement, and intra-observer agreement were assessed using the κ statistic.

Histopathological changes associated with CT-identified fissures were assessed by comparing the scoring between locations with and without genuine fissures (CT-identified fissures and verified with histological examination) using Mann–Whitney U test. *p* value < 0.05 was considered statistically significant. Agreement was considered slight, fair, moderate, substantial, and excellent if kappa was <0.20, 0.21–0.40, 0.41–0.60, 0.61–0.80, and >0.80 [31].

The distribution and imaging features of fissures on CT/MRI images and the associated surrounding bone pathology identified by the MRI were assessed with descriptive analysis.

## 3. Results

A lateral parasagittal groove and a sagittal groove were excluded from fissure assessment in this study due to the presence of a complete fracture.

Out of 291 locations, the total number of locations with comparable imaging and histology data for statistical analysis and assessment of fissures was 250 locations for both CT and histology, 245 locations for MRI-1 and histology, and 217 locations for MRI-2 and histology.

### 3.1. Identification of Fissures between Imaging Modalities (Supplemtary Table S1)

With histopathological findings as a gold standard, CBCT showed 88.5% sensitivity (CI95 = 82–94%), 61.3% specificity (CI95 = 53–69%), and a moderate agreement (κ = 0.48, *p* < 0.001); FBCT showed 84.1% sensitivity (CI95 = 77–90%), 72.3% specificity (CI95 = 64–79%), and a moderate agreement (κ = 0.56, *p* < 0.001); MRI-1 showed a 43.6% sensitivity (CI95 = 35–53%), 85.2% specificity (CI95 = 79–91%), and a fair agreement (κ = 0.30, *p* < 0.001); and MRI-2 showed a 37.6% sensitivity (CI95 = 28–48%), 87.1% specificity (CI95 = 81–92%), and a fair agreement (κ = 0.26, *p* < 0.001) for identifying fissures.

There was a substantial intra-observer agreement (κ = 0.67–0.8, *p* < 0.001) in all modalities and a substantial inter-modality agreement (κ = 0.73, *p* < 0.001) between CBCT and FBCT.

### 3.2. Features of Fissures on CT Images

A total number of 92 locations were identified with fissures by both CT systems and were confirmed with histological examination. Four types of fissures were characterised in the 92 fissures identified by both CT systems. The distribution of different types of fissures in each location are summarised in Table 1.

#### 3.2.1. Hypoattenuating Linear Defects

**Figure 2:**
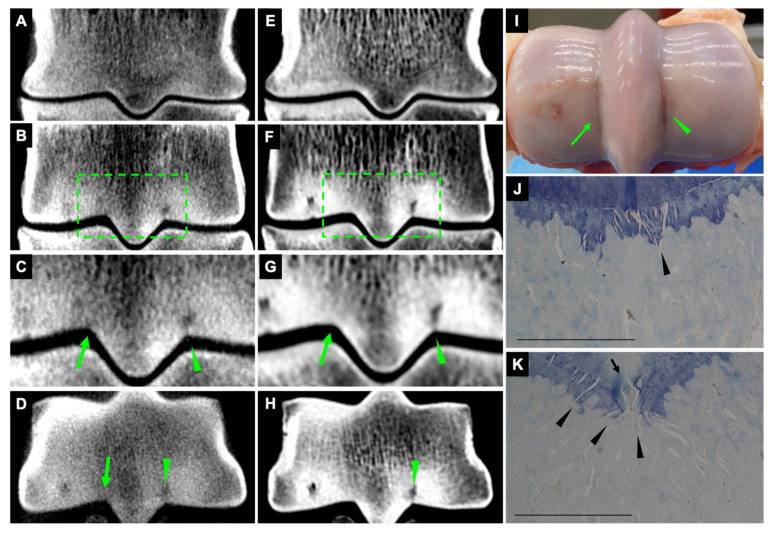
Fissures at the palmar aspect of the medial and lateral parasagittal grooves. Lateral is to the right. CBCT (**A**–**D**) and FBCT (**E**–**H**) dorsal and transverse orientation. Images (**A**,**E**) are diagrams showing the normal appearance of dorsal sections, without fissures in the parasagittal grooves. Images (**C**,**G**) are magnifications of the selected area on images (**B**,**F**). The medial parasagittal groove fissure appeared as striated hypoattenuating lines on the CBCT (arrow; (**C**,**D**)) and very subtle subchondral outline irregularity on the FBCT (arrow; (**G**)). The lateral parasagittal groove fissure appeared as hypoattenuating linear defects on both CBCT and FBCT (arrowhead; (**C**,**D**,**G**,**H**)). Macroscopic appearance of medial and lateral parasagittal groove fissures with articular cartilage defects ((**I**); arrow and arrowhead). Microscopic appearance of fissures at the medial (**J**) and lateral (**K**) parasagittal grooves. Microcracks in the calcified cartilage and subchondral bone plate appeared as oblique striated linear lesions and some were coalescing (arrowhead; (**J**,**K**)). Calcified cartilage cleft (arrow). Scale bar = 1 mm.

On CBCT and FBCT images, 21 fissures were identified as hypoattenuating linear defects in the subchondral bone in dorsal and transverse orientations (Figure 2).

#### 3.2.2. Striated Hypoattenuating Lines on CBCT and FBCT

**Figure 3:**
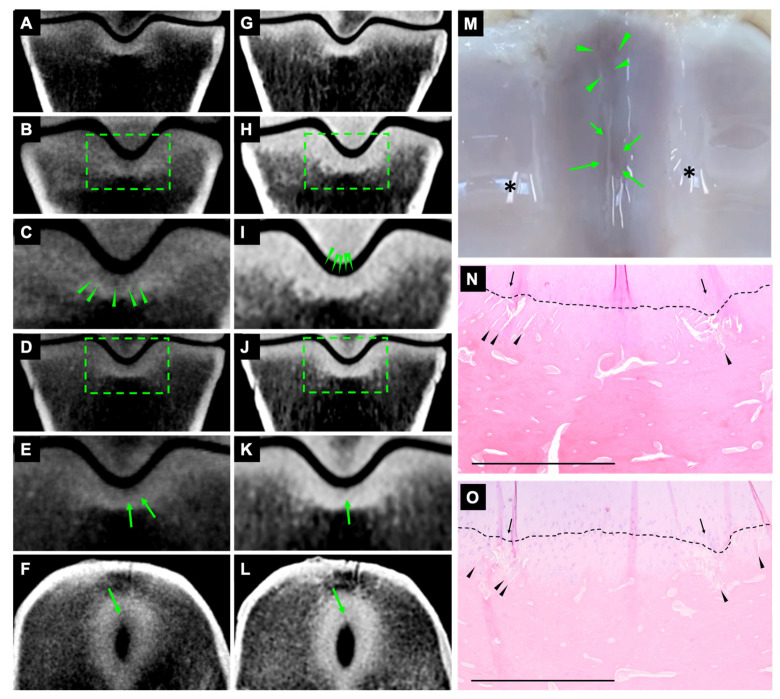
Fissures at the dorsal and middle aspects of the sagittal groove. Lateral is to the right. CBCT (**A**–**F**) and FBCT (**G**–**L**) dorsal and transverse orientation. Images (**A**,**G**) are diagrams showing the normal appearance of dorsal sections, without fissures in the sagittal groove. Images (**C**,**I**) are magnifications of the selected area on images (**B**,**H**). Striated hypoattenuating lines on CBCT and subchondral outline irregularity on FBCT at the dorsal aspect of sagittal groove (arrowheads; (**C**,**I**)). Images (**E**,**K**) are magnifications of the selected area on images (**D**,**J**). Striated hypoattenuating lines in the subchondral bone on CBCT and FBCT at the middle aspect of sagittal groove (arrow; (**E**,**K**)). A subtle, hypoattenuating line was seen in the transverse orientation (arrow; (**F**,**L**)). Macroscopic appearance of fissures at the dorsal and middle aspects of sagittal groove with subchondral discolouration (arrowheads and arrows; (**M**)). Artefacts of light reflection (*). Microscopic appearance of fissures at the dorsal (**N**) and middle (**O**) aspects of sagittal groove. Microcracks in the calcified cartilage and subchondral bone plate appeared as oblique striated linear lesions and some were coalescing (arrowhead; (**N**,**O**)). Tidemark incongruence (arrow, (**N**,**O**)). Interface between articular hyaline cartilage and calcified cartilage (dotted lines, (**N**,**O**)). Scale bar = 1 mm.

On CBCT and FBCT images, 34 fissures were identified as subtle striated hypoattenuated lines in the subchondral bone (Figure 2 and Figure 3). The fissures were most clear on dorsal orientation and appeared as very subtle, hypoattenuating lines on transverse orientation (Figure 3).

#### 3.2.3. Subchondral Outline Irregularity on CBCT and FBCT

On CBCT and FBCT images, 19 fissures were identified as subchondral outline irregularity. The fissures were most clear on the dorsal orientation and might be observed on the transverse orientation.

#### 3.2.4. Striated Hypoattenuating Lines on CBCT and Subchondral Outline Irregularity on FBCT

Eighteen fissures were identified as striated hypoattenuating lines on CBCT images and as subchondral outline irregularity on FBCT images (Figure 2 and Figure 3). The fissures were most clear on the dorsal orientation and might be observed on the transverse orientation (Figure 2).

### 3.3. Length of Fissures on CT Images

The median and interquartile range of fissures identified as hypoattenuating linear defects and striated hypoattenuating lines were 1.7 ± 0.6 mm on CBCT and 1.9 ± 1.0 mm on FBCT.

### 3.4. Features of Fissures on MR Images

A total number of 50 locations were identified with fissures on MR images and confirmed with histological examination. Of the 50 locations affected with fissures, 28 locations had concurrent, focal STIR hyperintensity in the adjacent subchondral/trabecular bone and were confirmed to have homogeneous, eosinophilic exudate in the marrow spaces on histological examination.

**Figure 4:**
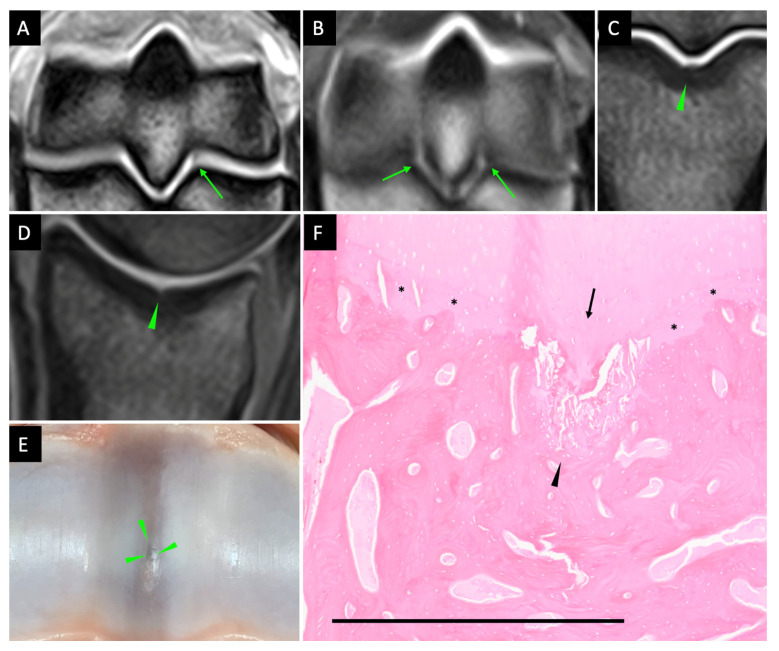
Parasagittal grooves and sagittal groove fissures on MR images. Lateral is to the right. Parasagittal groove fissures appeared as hypointense or hyperintense defects (arrow) on T1W 3D (**A**) and PD SE (**B**) transverse plane (the same lesions as Figure 2). Sagittal groove fissures appeared as subchondral bone irregularity (arrowhead) on the dorsal plane (**C**) and a hyperintense defect (arrowhead) on the sagittal plane (**D**) of the T1W 3D sequence. Macroscopic appearance of the fissure (**E**). Microscopic appearance of the sagittal groove fissure (**F**). Microcracks in the calcified cartilage appeared as coalesced oblique striated linear lesion (arrowhead). Tidemark incongruence (arrow) and variation in calcified cartilage depth (*). Scale bar = 1 mm.

Parasagittal groove fissures were identified as hyperintense or hypointense defects in the subchondral bone on all sequences and were most readily identified on the dorsal and transverse planes of T1W SE, PD SE, and T1W 3D sequences (Figure 4). Sagittal groove fissures were identified as an irregularity of the subchondral bone outline on the dorsal plane and a hyperintense defect on the sagittal plane (Figure 4). The distribution of MRI-identified fissures for each location and MRI sequence is summarised in Table 2.

### 3.5. Macroscopic Examination

Macroscopic abnormalities including articular cartilage erosion, partial-/full-thickness defects, and subchondral bone discolouration were found in the third metacarpal/metatarsal medial parasagittal groove at the palmar/plantar (*n* = 33), middle (*n* = 1), and dorsal (*n* = 18) aspects; the third metacarpal/metatarsal lateral parasagittal groove at the palmar/plantar (*n* = 32), middle (*n* = 1), and dorsal (*n* = 10) aspects; and the proximal phalanx sagittal groove at the palmar/plantar (*n* = 22), middle (*n* = 19), and dorsal (*n* = 19) aspects.

There was a moderate agreement (κ = 0.49, *p* < 0.001) between macroscopic and microscopic identification of fissures.

### 3.6. Histopathology Associated with Fissures (Supplemtary Table S2)

Out of 291 locations, fissures as microcracks in the calcified cartilage and subchondral bone (*n* = 114) were identified in the third metacarpal/metatarsal medial parasagittal groove at the palmar/plantar (*n* = 26), middle (*n* = 1), and dorsal (*n* = 3) aspects; the third metacarpal/metatarsal lateral parasagittal groove at the palmar/plantar (*n* = 22), middle (*n* = 0), and dorsal (*n* = 2) aspects; and the proximal phalanx sagittal groove at the palmar/plantar (*n* = 15), middle (*n* = 27), and dorsal (*n* = 18) aspects. One hundred and thirty-eight locations were considered free of fissures.

The scoring of histopathological changes was compared between true-positive diagnosis (*n* = 92) and true-negative diagnosis (*n* = 106) of fissures with both CBCT and FBCT.

The results showed that the scores of 7/8 histopathological changes in subchondral/trabecular bone were highly significant and more severe in locations with fissures. The observed sclerosis, trabeculae thickening, increased compact bone, increased osteon, subchondral bone collapse, microcracks, and increased woven bone indicated an increased bone remodelling and microdamage. The score of Howship’s lacunae was also significantly higher, indicating increased bone resorption. The scores of 3/5 histopathological changes in calcified cartilage were highly significant and more severe in locations with fissures. The observed tidemark incongruence, calcified cartilage depth variation, and vascular invasion indicated increased calcified cartilage and subchondral bone remodelling and neovascularisation. The score for calcified cartilage cleft was also significantly higher, indicating advancement of subchondral bone and/or articular cartilage injury. The scores of 2/7 histopathological changes in hyaline cartilage were highly significant and more severe in locations with fissures. The observed chondrocyte distribution and clustering indicated abnormal mechanical loading and extracellular matrix homeostasis. The scores of cartilage stain, cartilage thickness, and chondrocyte loss were also significantly higher, indicating abnormal extracellular matrix homeostasis and matrix degradation. Details of the comparison of histopathology scoring are listed in Table 3.

## 4. Discussion

The imaging features of third metacarpal/tarsal parasagittal groove and proximal phalanx sagittal groove fissures on CBCT, FBCT, and MR images have been described in this study, and the detection of fissures has been compared between imaging modalities using microscopic findings as the gold standard. The results partially support our first hypothesis that both CT systems would be superior to the low-field MRI in identifying fissures; however, the results did not show a superiority in FBCT over CBCT in detecting fissures. Histopathological features associated with fissures were assessed, and the results support our second hypothesis that the MRI was able to identify bone pathology adjacent to fissures by showing increased fluid signal, which was confirmed by the finding of homogeneous, eosinophilic exudate in the marrow spaces on histology. This may reflect the pathological status of fissures including inflammation or serous atrophy of the marrow fat [32]. The results of the histopathological examination also support our third hypothesis that histopathological features of fatigue injuries including subchondral bone sclerosis, microcracks, remodelling, and collapse are highly significantly associated with fissures.

There was a variation in the characteristics of fissures on CBCT and FBCT images in this study. Various features of third metacarpal parasagittal groove fissures have been reported in a previous equine cadaver limb study using a 64-slice FBCT [14]. These included a well-defined hypoattenuating lesion in the subchondral bone and a smaller area of hypoattenuation in the subchondral bone plate, which corresponded to the Type i and Type ii fissures identified in our study. The two different features of fissures were characterised by both CBCT and FBCT in our study in the third metacarpal/tarsal parasagittal groove and the proximal phalanx sagittal groove. Additionally, our study characterised another feature of fissures on both CBCT and FBCT, which was an irregularity of the subchondral bone outline (Type iii). This suggests that awareness of the different appearances of fissures may be important for image interpretation.

In total, 18 out of 92 (19.6%) fissures showed a difference in the characteristics of fissures between CBCT and FBCT in this study. The fissures appeared as striated hypoattenuated lines on the CBCT but as subchondral bone outline irregularity on the FBCT (Type iv). The difference in the appearance of fissures between CBCT and FBCT could result from a difference in CT image reconstruction. In human imaging studies, CBCT imaging has been suggested to have superior spatial resolution over FBCT imaging due to differences in instrumentation factors such as the detector and the reconstruction filter [33,34,35,36,37,38]. The reconstruction filter will always decrease spatial resolution with increased filtering to reduce the noise, and vice versa. The appearances of smaller or subtle lesions may be more affected by the difference in spatial resolution and signal-to-noise ratio between CBCT and FBCT, resulting in the hypoattenuating striations on CBCT images but subchondral outline irregularity on FBCT images.

The low-field MRI was markedly inferior to the CT systems in detecting the proximal phalanx sagittal groove fissures in this study. Forty-seven locations in the proximal phalanx sagittal groove were identified with fissures by both CT systems in the 33 cadaver limbs, while only three locations were detected with fissures by the MRI. In contrast, fissures in the third metacarpal/tarsal parasagittal groove were generally equally identified by both the MRI and the CT systems. The difference could result from the different anatomical locations and/or the direction of curvature outline between the proximal phalanx sagittal groove and the third metacarpal/tarsal parasagittal groove. Use of different MR imaging planes was helpful in assessing the areas of interest. An imaging plane 30° clockwise to the longitudinal axis of the third metacarpal/tarsal bone optimised assessment of the third metacarpal/tarsal parasagittal groove, while the proximal phalanx sagittal groove was not specifically emphasised by either a 30° clockwise or a 30° anti-clockwise imaging plane in this study. Therefore, special imaging techniques to improve the MRI detection of proximal phalanx sagittal groove fissures such as specific imaging angles or MR arthrography with intra-articular contrast may need further investigation.

The PD SE and T1W SE sequences were generally superior to the T1W 3D HR sequence, which was itself generally superior to the T2W FSE, T1W GRE FAST, and T2*W GRE FAST sequences for detecting the third metacarpal/tarsal parasagittal groove fissures in this study. Reduced slice thickness increases the spatial resolution, which is important for detecting small structural changes like fissures; however, it also decreases the signal to noise ratio, which influences image quality and potentially therefore the detection of fissures [39,40]. The results of different diagnostic performance among the various pulse sequences utilised in this study showed that the balance between signal-to-noise ratio and diagnostic value for detecting third metacarpal/tarsal parasagittal groove fissures may be optimal with PD SE and T1W SE sequences. To the author’s knowledge, there has not been a report comparing the efficiency of different MRI sequences in detecting third metacarpal/tarsal parasagittal groove fissures. The reported diagnostic performance of different pulse sequences in this study may provide useful information for clinical sequence application when the practicality of balancing between image quality and image acquisition time needs to be considered.

When histology and CT images were compared, there was a greater association between the third metacarpal/tarsal parasagittal groove and proximal phalanx sagittal groove fissures identified on CT images and the subchondral/trabecular bone histopathology, followed by the calcified cartilage histopathology and articular hyaline cartilage histopathology. Subchondral bone sclerosis, microcracks, and collapse are indications of subchondral bone remodelling resulting from repetitive loading stress and bone fatigue in both horses, humans, and rodents’ studies and were found in significantly greater histopathological scores in CT-identified fissures than non-fissures [6,28,41,42,43,44,45,46]. In the calcified cartilage, neovascularisation and reduced calcified cartilage thickness were indications of osteochondral disruption, subchondral bone remodelling, and osteoarthritis in humans and rodents and were also found in significantly higher scores in CT-identified fissures [43,47]. In the articular hyaline cartilage, chondrocyte clustering was associated with overloading exercise and osteoarthritis in horses, rabbits, and rodents and was found in significantly higher scores in CT-identified fissures [48,49]. Considering the higher number of histopathological abnormalities observed in the subchondral/trabecular bone region compared to the superficial hyaline cartilage region, it may be reasonable to suggest that the third metacarpal/tarsal parasagittal groove and proximal phalanx sagittal groove fissures identified on CT images in this study could originate from the subchondral/trabecular bone and could indicate fatigue bone injuries.

One of the limitations of this study is the influence of motion artefact on the diagnostic value of standing CT and MR imaging for detecting third metacarpal/tarsal parasagittal groove and proximal phalanx sagittal groove fissures could not be assessed in this cadaver limb study. As reduced image quality due to motion and artefacts in standing CBCT imaging compared to FBCT imaging under general anaesthesia was reported in a previous study [22], it may be inevitable that the sensitivity of CT and MR imaging reported in this study can decrease when applied to imaging in standing sedated horses. However, cadaver limb study using pathological examination to verify imaging findings provided advantages including microscopic evidence relevant to imaging diagnosis and potential pathological indications of fissures identified on CT and MR images. Another limitation to this cadaver limb study was the lack of information on the association or risk of stress/fatigue fractures with fissures identified on CT and MR images. Focal changes in calcified cartilage and subchondral bone under micro-X-ray was observed in young Thoroughbred horses that underwent conditioning exercise but not race training, indicating the potential of self-annealing of these micro-changes [50]. However, by validating the fissures identified by standing CT and MR imaging in this study, future longitudinal studies could follow up on the development or resolution of fissures characterised in this study. The limitation of using histopathological findings of microcracks as a gold standard without additional supporting micro-CT or micro-X-ray examination is fully recognised in this study. However, histological examination provides the advantage of assessing histopathological changes in hyaline cartilage, calcified cartilage, and subchondral/trabecular bone, which may provide a broader picture of histopathological features relevant to fissures identified on CT and MRI. Finally, the study was based on convenient cadaver limbs from a small number of Thoroughbred horses, so the histopathological features identified associated with fissures may not be fully representative of the development of fissures and stress fractures in all Thoroughbred horses. However, the objective of this study was to describe imaging features of fissures and compare the identification of fissures between CBCT, FBCT, and low-field MRI. Histopathological features identified in this study could add further information to imaging interpretation of fissures.

## 5. Conclusions

In conclusion, the CBCT, FBCT, and low-field MRI utilised in this study had similar performance in detecting third metacarpal/tarsal parasagittal groove fissures. However, both CT systems were superior to MRI in detecting proximal phalanx sagittal groove fissures. Four types of appearances of fissures on CT images were characterised in this study: three were identical between CBCT and FBCT (i) hypoattenuating linear defects, (ii) striated hypoattenuating lines, and (iii) subchondral outline irregularity. The fourth type was different between CBCT and FBCT, which appeared as striated hypoattenuating lines on CBCT and subchondral outline irregularity on FBCT. On the low-field MRI, fissures were characterised as hyperintense or hypointense defects in the subchondral bone. PD SE, T1W SE, and T1W 3D HR sequences identified more fissures than other sequences. MRI also identified increased fluid signal in the subchondral/trabecular bone adjacent to fissures, which could indicate inflammation or serous atrophy and was not identified on CT images. Histopathological features of fatigue injuries including subchondral bone sclerosis, microcracks, and collapse were associated with CT-identified fissures, supporting the importance of the detection of fissures.

## Figures and Tables

**Table 1:** Distribution of four types of fissures identified with CBCT and FBCT. Type (i) hypoattenuated linear defects. Type (ii) striated hypoattenuating lines on CBCT and FBCT. Type (iii) subchondral outline irregularity on CBCT and FBCT. Type (iv) striated hypoattenuating lines on CBCT and subchondral outline irregularity on FBCT.

Locations	Both CT	Type (i)	Type (ii)	Type (iii)	Type (iv)
Medial parasagittal groove—palmar/plantar	24	12	8	3	1
Medial parasagittal groove—middle	0	0	0	0	0
Medial parasagittal groove—dorsal	0	0	0	0	0
Lateral parasagittal groove—palmar/plantar	21	6	10	3	2
Lateral parasagittal groove—middle	0	0	0	0	0
Lateral parasagittal groove—dorsal	0	0	0	0	0
Sagittal groove—palmar/plantar	6	1	1	2	2
Sagittal groove—middle	24	1	11	4	8
Sagittal groove—dorsal	17	1	4	7	5
Total counts	92	21	34	19	18

**Table 2:** Distribution of fissures identified by different MRI sequences. A: T1W 3D HR, B: T2W FSE, C: T2*W 3D, D: STIR FSE, E: PD SE, F: T1W SE, G: T1W GRE FAST, H: T2W FSE FAST, I: T2*W GRE FAST and, J: STIR FSE FAST sequences.

Locations	MRI	A	B	C	D	E	F	G	H	I	J
Medial parasagittal groove—palmar/plantar	25	14	11	8	6	14	17	9	6	10	1
Medial parasagittal groove—middle	0	0	0	0	0	0	0	0	0	0	0
Medial parasagittal groove—dorsal	1	1	1	1	0	1	1	1	0	1	0
Lateral parasagittal groove—palmar/plantar	21	7	4	5	3	13	12	7	1	7	1
Lateral parasagittal groove—middle	0	0	0	0	0	0	0	0	0	0	0
Lateral parasagittal groove—dorsal	0	0	0	0	0	0	0	0	0	0	0
Sagittal groove—palmar/plantar	0	0	0	0	0	0	0	0	0	0	0
Sagittal groove—middle	3	3	1	1	0	2	1	1	0	1	0
Sagittal groove—dorsal	0	0	0	0	0	0	0	0	0	0	0
Total counts	50	25	17	15	9	30	31	18	7	19	2

**Table 3:** Comparison of histopathological scoring between genuine fissures and non-fissures on CT images.

Items	*p* Value
Hyaline cartilage	
Reduced staining for glycosaminoglycans in cartilage	0.03 *
Cartilage surface irregularity	0.15
Cartilage fibrillation	0.73
Cartilage thickness variation	0.03 *
Irregular chondrocyte distribution	<0.001 **
Chondrocyte loss/necrosis	0.01 *
Chondrocyte clustering	<0.001 **
Calcified cartilage	
Tidemark incongruence	<0.001 **
Calcified cartilage cleft	0.03 *
Calcified cartilage depth variation	<0.001 **
Vascular invasion	<0.001 **
Island of hyaline cartilage in subchondral bone plate	0.03 ^†^
Subchondral/trabecular bone	
Sclerosis of subchondral bone plate and adjacent cancellous bone	<0.001 **
Subchondral bone collapse	<0.001 **
Replacement of cancellous bone with compact bone	<0.001 **
Trabecular thickening with reduced marrow spaces	<0.001 **
Replacement with osteon/lamellar bone	<0.001 **
Microcracks in cancellous bone	<0.001 **
Replacement with woven bone	<0.001 **
Howship’s lacunae with/without osteoclast	0.04 *

* Significant difference (true positive > true negative). ** Highly significant difference (true positive > true negative). ^†^ Significant difference (true negative > true positive).

## Data Availability

The data presented in this study are available from the corresponding author upon request. The data are not publicly available due to usage in a thesis.

## Supplementary Materials

The following supporting information can be downloaded at https://www.mdpi.com/article/10.3390/ani13182912/s1, Table S1: Details of fissure diagnosis; Table S2: Details of histopathological scoring of fissures.
