# Supplementary material for: Three-Dimensional Imaging and Histopathological Features of Third Metacarpal/Tarsal Parasagittal Groove and Proximal Phalanx Sagittal Groove Fissures in Thoroughbred Horses
Source: Animals (Basel). 2023 Sep 14;13(18):2912. doi: 10.3390/ani13182912 (PMC10525482; doi:10.3390/ani13182912)
Supplement: Supplementary file 1 [file animals-13-02912-s001.zip › Supplementary table 1.pdf]

Supplementary Table S1 Details of fissure diagnosis. CBCT (cone-beam CT). FBCT (fan-beam CT). MRI-1 (MRI sequence set 1). MRI-2 (MRI sequences set 2).

Number of fissures detected on imaging modalities compared with gold standard			
		Microcracks (gold standard)	
Imaging		Without	With
CBCT (diagnosis)	Without	84	13
	With	53	100
FBCT (diagnosis)	Without	99	18
	With	38	95
MRI-1 (diagnosis)	Without	115	62
	With	20	48
MRI-2 (diagnosis)	Without	108	58
	With	16	35
T1W 3D (detection)	Without	123	85
	With	11	25
T2W FSE (detection)	Without	122	93
	With	12	17
T2*W 3D (detection)	Without	125	95
	With	9	15
STIR FSE (detection)	Without	131	101
	With	3	9
PD SE (detection)	Without	120	80
	With	14	30
T1W SE (detection)	Without	120	79
	With	14	31
T1 GRE FAST (detection)	Without	111	75
	With	12	18
T2W FSE FAST (detection)	Without	116	86
	With	7	7
T2*W GRE FAST (detection)	Without	114	74
	With	9	19
STIR FSE FAST (detection)	Without	120	91
	With	3	2
