# Supplementary material for: Three-Dimensional Imaging and Histopathological Features of Third Metacarpal/Tarsal Parasagittal Groove and Proximal Phalanx Sagittal Groove Fissures in Thoroughbred Horses
Source: Animals (Basel). 2023 Sep 14;13(18):2912. doi: 10.3390/ani13182912 (PMC10525482; doi:10.3390/ani13182912)
Supplement: Supplementary file 1 [file animals-13-02912-s001.zip › Supplementary table 2.pdf]

Supplementary Table S2 Details of histopathological scoring of fissures. CBCT (cone-beam CT). FBCT (fan-beam CT).

Number of histopathological scoring of fissures			
		Microcracks/CBCT/FBCT	
Histopathological score		Without fissure	With fissure
Reduced staining for glycosaminoglycans in cartilage	0	29	15
	1	13	22
	2	12	14
	3	24	35
Cartilage surface irregularity	0	61	62
	1	14	6
	2	2	6
	3	1	12
Cartilage fibrillation	0	64	73
	1	12	7
	2	1	5
	3	1	1
Cartilage thickness variation	0	54	48
	1	19	21
	2	4	13
	3	1	4
Irregular chondrocyte distribution	0	26	9
	1	26	31
	2	20	19
	3	6	27
Chondrocyte loss/necrosis	0	11	8
	1	29	22
	2	28	31
	3	10	25
Chondrocyte clustering	0	22	8
	1	50	61
	2	4	11
	3	2	6
Tidemark incongruence	0	23	1
	1	41	42
	2	11	32
	3	2	10
Calcified cartilage cleft	0	76	77
	1	0	8
	2	0	0
	3	1	0
Calcified cartilage depth variation	0	36	9
	1	22	26
	2	6	21
	3	13	29

Vascular invasion	0	64	13
	1	6	32
	2	1	26
	3	6	14
Island of hyaline cartilage in subchondral bone plate	0	36	53
	1	27	24
	2	0	4
	3	14	4
Sclerosis of subchondral bone plate and adjacent cancellous bone	0	71	6
	1	20	48
	2	0	6
	3	0	32
Subchondral bone collapse	0	87	63
	1	4	25
	2	0	4
	3	0	0
Replacement of cancellous bone with compact bone	0	47	6
	1	32	23
	2	7	36
	3	1	26
Trabecular thickening with reduced marrow spaces	0	45	3
	1	30	20
	2	10	32
	3	2	35
Replacement with osteon/lamellar bone	0	34	1
	1	44	18
	2	11	20
	3	1	53
Microcracks in cancellous bone	0	35	12
	1	36	22
	2	12	31
	3	3	26
Replacement with woven bone	0	52	27
	1	30	46
	2	8	18
	3	0	0
Howship’s lacunae with/without osteoclast	0	20	31
	1	43	39
	2	15	18
	3	12	3
